# Baseline-Free Adaptive Crack Localization for Operating Stepped Rotors Based on Multiscale Data Fusion

**DOI:** 10.3390/s20195693

**Published:** 2020-10-06

**Authors:** Zhiwen Lu, Shancheng Cao, Rui Yuan, Yong Lv

**Affiliations:** 1 Hubei Key Laboratory of Mechanical Transmission and Manufacturing Engineering, Wuhan University of Science and Technology, Wuhan 430081, China; luzhiwen@wust.edu.cn (Z.L.); yuanrui@wust.edu.cn (R.Y.); 2 The Key Laboratory of Metallurgical Equipment and Control of Education Ministry, Wuhan University of Science and Technology, Wuhan 430081, China; 3 School of Astronautics, Northwestern Polytechnical University, Xi’an 710072, China; shancheng.cao@nwpu.edu.cn

**Keywords:** Crack localization, rotors, nonlinear, D-S evidence fusion, multiscale, super-harmonic characteristic deflection shape

## Abstract

Crack localization in running rotors is very important and full of challenges for machinery operation and maintenance. Characteristic deflection shapes or their derivatives based methods seem to be promising for crack localization in rotors. Despite the substantial advantages, several critical issues still need to be addressed to enhance the efficiency of this kind of method for practical applications. Two problems are considered in this work: 1. How to localize single or multiple cracks accurately avoiding the interference of commonly existing steps without baseline information on pristine rotors; 2. How to improve the crack localization performance under a noisy environment. To circumvent the issues, a novel baseline-free adaptive crack localization method is proposed based on data fusion of multiscale super-harmonic characteristic deflection shapes (SCDSs). In this method, crack induced asymmetry and nonlinearity of crack breathing are utilized to simultaneously eliminate the interference from the steps without a reference model. To enhance the noise robustness, the multiscale representations of SCDSs are made in Gaussian multiscale space, and Teager energy operator is applied to the multiscale SCDSs to amplify the crack induced singularities and construct the multiscale Teager super-harmonic characteristic deflection shapes (TSCDSs). Moreover, fractal dimension is designed as an evaluator to select the proper multiscale TSCDSs for data fusion adaptively. Then, a new damage index is derived for crack localization by Dempster-Shafer’s (D-S) evidence fusion of the adaptively selected multiscale TSCDSs. Finally, the feasibility and the effectiveness are verified by both numerical and experimental investigations.

## 1. Introduction

Crack monitoring and diagnosis in rotors based on vibration signals has been widely investigated and is an important field in the structural health monitoring (SHM) of rotating machines. According to the level of identification attempted, the relevant studies could be classified into four levels [1], which are crack detection [2,3,4,5,6,7,8,9,10,11,12,13], crack localization [14,15,16,17], crack identification [18,19,20,21] and remaining useful life prediction [22,23,24]. Among the four levels, many studies have already been conducted on crack detection. As for crack localization in rotors, it is of great importance for the maintenance of rotating machines, however, fewer studies have been carried out and it is still difficult to apply in engineering. This work aims at developing a crack localization method for rotors containing commonly existing steps under operating and noisy conditions.

For crack localization, it is necessary to have enough spatial information. Crack localization by modal parameters, such as natural frequencies and mode shapes or their derivatives, has been widely investigated in structures [25,26,27,28]. As the natural frequencies are global parameters, reference models are required to provide more spatial information for the natural frequency-based methods. For the mode shape-based method, it contains enough spatial information and has been applied in stationery structures successfully, but it is difficult to obtain mode shapes for rotors under operating conditions. Besides, mode shapes only involve linear information, which cannot deal with nonlinear cases such as rotors with fatigue breathing cracks, or it will omit some helpful nonlinear information. Hence, mode shape-based crack localization methods are not suitable for operating rotors.

Similar with the mode shape, a more general concept called the characteristic deflection shape (CDS) was proposed by Cao and Ouyang [29] to localize cracks for structures. The CDS is defined as an intrinsic spatial data structure extracted from multiple-sensor signals following a certain rule [30]. According to the definition, the CDS includes but is not limited to a mode shape or an operational deflection shape (ODS) [31], and thus, it will be suitable for rotating rotors. Saravanan et al. [32] defined a kind of CDS with the kurtosis of ODS, which was utilized to realize crack localization in running rotors. A form of CDS called the amplitude deviation curve was proposed by Babu et al. [33], in which the discontinuities were extracted to indicate the crack locations in a rotor. Those methods are effective for serious linear damage in structures. However, for fatigue cracks with nonlinear breathing behavior, how to improve the localization performance still needs further investigation.

From this aspect, Prawin et al. [34] developed a breathing crack localization method by utilizing the crack-induced nonlinear components based on the spatial curvature derived from the high order frequency component from distributed sensors. Broda et al. [35] utilized the ratio of a super-harmonic frequency to the fundamental harmonic frequency at different measurement locations for breathing crack localization. Lu et al. [36] realized breathing cracks localization in rotors by using the breathing crack induced super-harmonics to extract the CDS at high order frequencies. Moreover, a detailed literature review was presented by Bovsunovsky and Surace [37] for breathing crack identification in structures. These studies have shown that the CDS at higher order frequency could be more helpful for crack localization of cracks performing nonlinear behaviors.

Cracks in rotors of critical machines are the most dangerous faults. The occurrence of cracks will lead to local stiffness reduction of the rotor which will generate discontinuities or distortions at crack locations. The commonly existing steps in shafts will also cause discontinuities or distortions, which will affect the crack localization. The localization results could be misguided, if there is no baseline model of the rotor or prior location information of the steps. However, one key point we should note is that the cracks in rotors are usually fatigue cracks, which cyclically open and close (also called breathing) with the rotation of rotors, which make the system nonlinear. Therefore, the crack breathing phenomenon is a unique feature of fatigue cracks. If crack induced nonlinearity and stiffness reduction could be explored simultaneously, cracks in stepped rotors could be localized exclusively.

Utilizing the distinct feature between nonlinear cracks and linear steps, a crack localization method based on super-harmonic characteristic deflection shapes (SCDSs) was proposed by the authors [36]. In that work, local shape distortions induced by cracks in the SCDSs were extracted to indicate the crack locations in stepped rotors. However, because of the weakness of the crack and the heavy noise in the obtained signals, this work still requires further investigation for crack localization to improve the noise robustness.

To circumvent the noise-robustness issue, a Bayesian fusion method was proposed for crack localization using the SCDSs in multiscale space by the authors [38]. However, a priori probability is needed by Bayesian fusion theory, and how to select the proper scale parameters also needs further study. Compared with Bayesian fusion theory [39,40], the Dempster-Shafer’s (D-S) evidence theory is a mathematical approach to make inferences based on imprecise and uncertain information, which is derived from different information sources, and no priori probability is needed.

In view of the aforementioned issues, a novel baseline-free adaptive crack localization method for operating stepped rotors based on D-S evidence fusion of multiscale SCDSs is developed. To address the robustness issue, Gaussian multiscale analysis is adopted to transform the SCDSs into multiscale SCDSs, and Teager energy operator is applied to the multiscale SCDSs to obtain the multiscale Teager super-harmonic characteristic deflection shapes (TSCDSs), which enhances the weak singularities that are induced by cracks. Furthermore, a fractal dimension is proposed and designed as an evaluator to adaptively select the proper multiscale TSCDSs. After this, the selected proper multiscale TSCDSs are utilized to construct a damage index by D-S evidence fusion. Finally, numerical simulations and rig experiments are performed to show the performance of the proposed method.

The main contributions of the work are in the following:

1. A novel baseline-free adaptive crack localization method for rotating stepped rotors with single or multiple cracks is developed and validated numerically and experimentally.
2. A novel damage index based on data fusion of multiscale TSCDSs with D-S evidence theory is derived to overcome the interference of steps and improve the robustness of the crack location method.
3. The fractal dimension is designed as an evaluator to adaptively select the optimal multiscale TSCDSs for data fusion.

The remainder of the paper is arranged as follows. Detail presentation of the proposed method is introduced in Section 2. Then, numerical investigations are performed for cracked and stepped rotors in Section 3. Experimental validation is carried out in Section 4. Discussions about the influence of scales and comparison with a previous single-scale method are performed in Section 5. Finally, conclusions are drawn.

## 2. Method Description

### 2.1. SCDS

The proposed SCDS is a spatial shape of multi-sensor signals at a super-harmonic frequency which can be extracted by using frequency domain decomposition (FDD) [36,41]. The details are as follows.

First, the system response matrix Y is assembled by the simultaneously measured responses from sensors distributed along the shaft, which can be expressed as:

(1) $$Y=\left[y_{1},\ldots ,y_{n}\right]=\left[\begin{array}{ccc} y_{11} & \ldots & y_{1n}\\ \ldots & \ldots & \ldots \\ y_{l1} & \ldots & y_{ln} \end{array}\right]\in {\mathbb{R}}^{l\times n}$$

where $y_{i}$ is the response from sensor *i*, *l* is the length of each response, and *n* is the number of sensors.

After obtaining the response matrix, its corresponding correlation matrix $R_{\mathrm{yy}}$ can be computed by:

(2) $$R_{\mathrm{yy}}(\tau )=\left[\begin{array}{ccc} R_{11} & \cdots & R_{1n}\\ \vdots & R_{ij} & \vdots \\ R_{n1} & \cdots & R_{nn} \end{array}\right]\in {\mathbb{R}}^{n\times n}$$

where $R_{ij}$ is the correlation function between $y_{i}$ and $y_{j}$, which can be expressed as:

(3) $$R_{ij}(\tau )=E\left[y_{i}(t)y_{j}(t+\tau )\right]$$

here E is the averaging operator and τ is the time-lag.

Then the power spectral density matrix $G_{\mathrm{yy}}$ can be obtained by the Fourier transform of the correlation matrix as:

(4) $$G_{\mathrm{yy}}\left(\omega \right)=F\left[R_{\mathrm{yy}}(\tau )\right]\in {\mathbb{R}}^{n\times n}$$

From the expression of $R_{\mathrm{yy}}$, it can be derived that the power spectral density matrix $G_{\mathrm{yy}}$ is square and positive definite. Then, $G_{\mathrm{yy}}$ at frequency $\omega_{i}$ can be expressed by singular value decomposition as:

(5) $$G_{\mathrm{yy}}\left(\omega_{i}\right)=U_{i}S_{i}U_{i}{}^{T}$$

where ${\left(U_{i}\right)}_{n\times n}=\left[u_{i1},\ldots ,u_{in}\right]$ is an orthogonal matrix containing the singular vectors $u_{ij}$; ${\left(S_{i}\right)}_{n\times n}$ is a diagonal matrix with singular values at the diagonal entries.

$u_{i1}$ is the first singular vector of $G_{\mathrm{yy}}$ at frequency $\omega_{i}$, which is the dominant mode of the structure at frequency $\omega_{i}$ [41]. Thus, $u_{i1}$ can be taken as a dominant feature of all measured responses at frequency $\omega_{i}$ and its shape is defined as a characteristic deflection shape (CDS). Furthermore, if $\omega_{i}$ is a super-harmonic frequency, the corresponding CDS is called a super-harmonic CDS (SCDS) [36].

For cracked rotors, nonlinear super-harmonic components induced by the breathing and asymmetry of cracks are recognized as common practice. Hence, the extracted SCDSs contain both the crack induced nonlinearity and stiffness reduction, which could be useful to eliminate the interference from steps, as the steps are linear and there is no nonlinear super-harmonics generated by them.

### 2.2. Multiscale SCDS

Heavy noise contamination is the common case in rotating machines. To make the SCDS-based crack localization method more robust to noise, the multiscale representations of SCDSs are made by Gaussian multiscale analysis (GMA). According to GMA, the multiscale SCDS can be expressed as the convolution between Gaussian kernel G and the extracted SCDS ϕ:

(6) L(x,σ)=G(x,σ)⊗ϕ(x)

where x(x=1,2,…,n) is the location of the measurement point, σ is the scale parameter, the Gaussian kernel G can be expressed as:

(7) $$G\left(x,\sigma \right)=\frac{1}{2\pi \sigma^{2}}e^{-\frac{x^{2}}{2\sigma^{2}}}$$

As the crack induces weak singularities in the SCDSs, the Teager energy operator (TEO) is applied to the obtained multiscale SCDS to generate the multiscale TSCDS, which is defined as [38]:

(8) $$T\left(x,\sigma \right)=L^{2}\left(x,\sigma \right)-L\left(x-1,\sigma \right)L\left(x+1,\sigma \right)$$

### 2.3. Adaptive Selection of Proper Scales

The crack sensitivity is different for various scale parameters, thus how to select the proper scales is important. If the selected scales are too large, the corresponding multiscale SCDSs will be too smooth. In this case, both the noise and the crack induced singularity are filtered out. While if the selected scales are too small, the de-noising performance will be not satisfied. The manual scale selection method requires many trial and errors, and the transferability is low when using the same scale parameters for different cases. Therefore, in order to enhance the transferability and robustness of the multiscale crack localization method, an adaptive scale selection method is proposed. The key point is to establish an evaluation index (EI) to evaluate the selected multiscale TSCDS automatically. Here a fractal dimension (FD) [42] scale selector is designed for this purpose. FD is a kind of measurement for complexity. The higher the value, the more complex the curve. As the crack induced singularities or distortions will make the SCDSs more complex at the crack positions, the FD at every measurement point can indicate the probability of a crack. Thus the FD peaks of all measurement points indicate the crack locations of cracks in one scale. For all scales, the larger the FD peak in one scale, then potentially, the higher the localization performance. By setting the required scale number, the multiscale TSCDSs can be selected adaptively.

The FD of a curve with *n* points $\left(O_{1},\ldots ,O_{n}\right)$ is defined by [42]:

(9) $$FD=\frac{\mathrm{lg}\left(n-1\right)}{\mathrm{lg}(n-1)+\mathrm{lg}(d/L)}$$

(10) $$d=\max_{2\leq i\leq n}\mathrm{dist}(O_{1},O_{i})$$

(11) $$L=\sum_{i=1}^{n-1}\mathrm{dist}(O_{i},O_{i+1})$$

where, dist(⋅,⋅) represents the distance between two points.

In order to show the local complexity of a multiscale TSCDS, a sliding window with width of *W* is applied to truncate the curve, and with a sliding step of one point. Then the FD in the *j*th window of a multiscale TSCDS with a scale of σ can be expressed as:

(12) $$FD\left(j,\sigma \right)=\frac{\mathrm{lg}\left(W-1\right)}{\mathrm{lg}(W-1)+\mathrm{lg}(d\left(j\right)/L\left(j\right))}$$

(13) $$d\left(j\right)=\max_{j\leq q\leq j+W-1}\mathrm{dist}(O_{j},O_{q})$$

(14) $$L\left(j\right)=\sum_{q=j}^{j+W-2}\mathrm{dist}(O_{q},O_{q+1})$$

where *j* is the midpoint position in the window.

In order to realize scale selection adaptively, an evaluation index (EI) must be defined to judge if the current scale is satisfied. The EI of the multiscale TSCDS with scale σ is defined as:

(15) $$EI\left(\sigma \right)=\frac{\left(n-1\right)\max_{1\leq j\leq n}FD\left(j,\sigma \right)}{\sum_{j=1}^{n}FD\left(j,\sigma \right)-\max_{1\leq j\leq n}FD\left(j,\sigma \right)}$$

From the expression of EI, one can see that when the local complexity of the multiscale TSCDS is higher and the mean complexity of the rest curve is lower, then the EI will be larger and the corresponding scale is more possible to be selected, which satisfies the scale selection requirement. After obtaining the EIs of all scales, by sorting them from large to small, then, the multiscale TSCDSs with larger evaluation index values are selected automatically for data fusion.

### 2.4. A Novel Damage Index

In the following, a novel baseline-free and robust damage index is derived by fusing the multiscale TSCDS based on D-S evidence theory [39].

Let a finite set θ have mutually exclusive and exhaustive propositions:

(16) $$\theta =\left\{e_{1},e_{2},\ldots ,e_{n}\right\}$$

where $e_{i}\left(i=1,2,\ldots n\right)$ represents the n measurement points used to obtain the responses, which corresponds to the locations in rotors.

Then the basic probability assignment (BPA) is defined by a function $m:2^{\theta }\to \left[0,1\right]$, which satisfies:

(17) 0≤m(A)≤1

(18) m(ϕ)=0

(19) $$\sum_{A\subseteq \theta }m\left(A\right)=1$$

where A denotes any subset of $2^{\theta }$, ∅ is an empty set, and m(A) is the BPA function of the proposition A.

In this work, the probability of a crack which occurs at location $x_{i}$ (*i* = 1, 2…, *n*) in the *k*th scale is expressed as Equation (20):

(20) $$m_{k}^{\sigma }\left(S_{k}\right)=\frac{T\left(x_{i},\sigma_{k}\right)}{\sum_{j=1}^{n}T\left(x_{j},\sigma_{k}\right)}$$

From the equation, one can see that the value of $T\left(x_{i},\sigma_{k}\right)$ indicates the probability that a crack locates at $x_{i}$ under the *k*th scale.

Then, the BPAs can be fused by the D-S evidence combination rule. In this work, information sources are defined as the multiscale TSCDS with any scale parameter from *S*_1_ to *S*_M_, where, *M* is the number of information sources used for fusion corresponding to the scale numbers, and $m_{k}^{\sigma }\left(S_{k}\right)\left(k=1,2,\ldots ,M\right)$ are the BPAs corresponding to the *M* sources.

Here, the combination rule of the first two data sources *S*_1_ and *S*_2_ can be expressed as follows:

(21) $$m_{1}^{\sigma }\left(C_{1}\right)=\frac{1}{1-K_{1}}\sum_{S_{1}\cap S_{2}=C_{1}}m_{1}^{\sigma }\left(S_{1}\right)m_{2}^{\sigma }\left(S_{2}\right)$$

where $K_{1}$ represents the weight of conflict between evidence 1 and 2, which is expressed as:

(22) $$K_{1}=\sum_{S_{1}\cap S_{2}=\phi }m_{1}^{\sigma }\left(S_{1}\right)m_{2}^{\sigma }\left(S_{2}\right)$$

when $K_{1}=1$, $m_{1}^{\sigma }\left(C_{1}\right)$ does not exist, which means that the two sources *S*_1_ and *S*_2_, are in full contradiction.

Then, fuse the remaining sources in the same way, which can be summarized as:

(23) $$m_{M-1}^{\sigma }\left(C_{M-1}\right)=\frac{1}{1-K_{M-1}}\sum_{C_{M-2}\cap S_{M}=C_{M-1}}m_{M-2}^{\sigma }\left(C_{M-2}\right)m_{M}^{\sigma }\left(S_{M}\right)$$

(24) $$K_{M-1}=\sum_{C_{M-2}\cap S_{M}=\phi }m_{M-2}^{\sigma }\left(C_{M-2}\right)m_{M}^{\sigma }\left(S_{M}\right)$$

### 2.5. General Procedure of the Proposed Crack Localization Method

**Figure 1:**
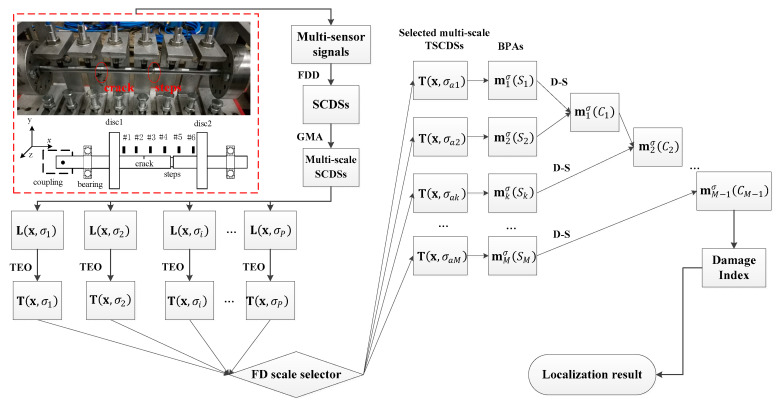
General procedure of the proposed crack localization method.

According to the above descriptions, the general procedure of the proposed baseline-free adaptive crack localization method is summarized in Figure 1. The main steps are as follows.

Step1: Obtain the vertical responses of the investigated rotors via distributed measurement using multiple sensors, and assemble the response matrix;
Step2: Extract the SCDSs by frequency domain decomposition, and select the sensitive SCDS to avoid the interference of the steps in shafts;
Step3: Obtain the multiscale TSCDSs $T\left(x,\sigma_{i}\right)\left(i=1,2,\ldots ,P\right)$, where, P is the total scale numbers) by using GMA and TEO;
Step4: Adaptively select the optimal multiscale TSCDSs ($T\left(x,\sigma_{ak}\right)\left(k=1,2,\ldots ,M\right)$, where, M is the selected scale numbers) by FD scale selector;
Step5: Calculate the BPAs ($m_{k}^{\sigma }\left(S_{k}\right)\left(k=1,2,\ldots ,M\right)$) of the adaptively selected multiscale TSCDSs;
Step6: Fuse the BPAs one by one using the D-S combination rule;
Step7: Calculate the damage indexes of every measurement point, of which the peaks indicate the locations of cracks.

## 3. Numerical Investigation

### 3.1. Numerical Model of a Stepped Rotor with Cracks

**Figure 2:**
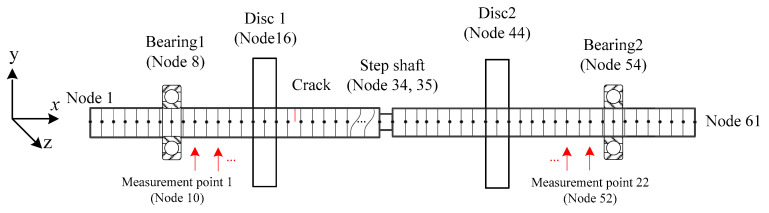
Schematic diagram of the numerical rotor and its finite element model.

To study the performance of the proposed method, a numerical rotor with its finite element model shown in Figure 2 is used, which had been modeled by the authors in [36,38]. To model the shaft, two-node Timoshenko beam elements with six degrees-of-freedom (DOFs) of each node were utilized to discretize the shaft into 60 equivalent elements. The discs were modeled as six DOFs lumped inertias which were added to the corresponding nodes. The bearings were simplified as isotropic spring-damping systems. Fixed constrains were applied to the driving end of the rotor at the torsional and axial DOFs. The step shaft was modeled by reducing the diameter of the shaft. The transverse cracks were considered and modeled by crack elements considering crack breathing phenomena, but no crack propagating during the measurement of responses, where the crack stiffness matrix of a crack was derived from linear fracture mechanics with the Crack Closure Line Position (CCLP) method [43]. By assembling all of the element matrices, the rotor motion equation was established in the stationary coordinate system as [36]:

(25) $$M\ddot{q}+\left(D+\Omega D_{g}\right)\dot{q}+K\left(t\right)q=F_{u}+F_{g}+F_{ex}$$

where q is the displacement vector, M, D, Ω, $D_{g}$, K(t) is the system mass matrix, system Rayleigh damping matrix, rotating frequency, system gyroscopic matrix, system stiffness matrix, respectively, and $F_{u}$, $F_{g}$, $F_{ex}$ is the unbalance excitation force vector, gravitational excitation force vector, and external excitation force vector, respectively.

The established motion Equation (25) is time-varying and state-dependent because of crack breathing. In order to solve this nonlinear equation and obtain the responses, the Newmark method [44] is adopted. To capture the fast-varying breathing behavior and consider the computation complexity, the sampling frequency is set as 5000 Hz. Rotating speed in the simulations remains at 840 r/min, about one-third of the first critical rotating speed which has been verified by a modal experiment, to take the 2 × frequency component away from the critical rotating frequency and to make its amplitude observable [45]. A total of 22 measurement points are set along the shaft between the bearings from node 10 to node 52 every two nodes in the vertical direction, as shown in Figure 2. In order to evaluate the proposed method, three typical rotors are simulated, of which the positions of cracks and steps are shown in Table 1. All the cracks considered in the simulations are with the depth of 0.15*D*, where *D* is the diameter of the shaft.

### 3.2. Localization Results

**Figure 3:**
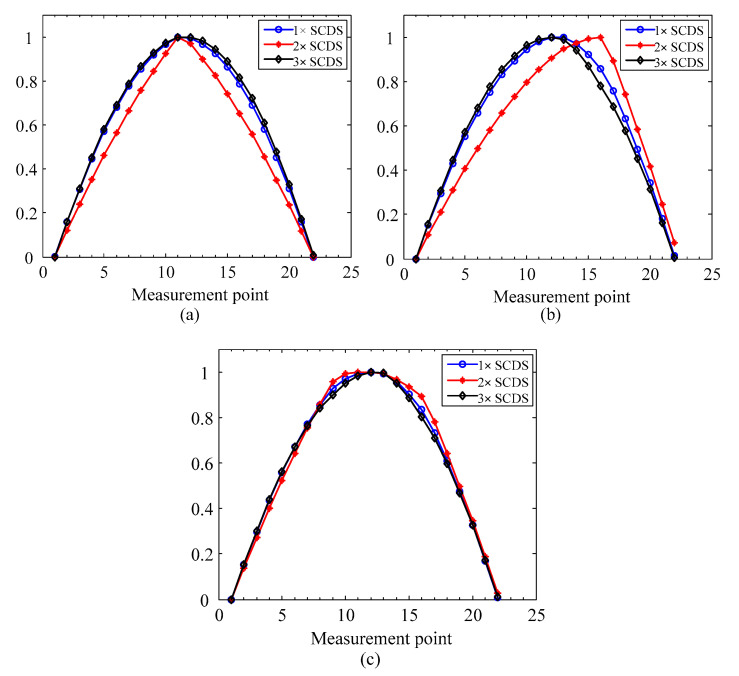
The first three super-harmonic characteristic deflection shapes (SCDSs): (**a**) simulation 1; (**b**) simulation 2; (**c**) simulation 3.

**Figure 4:**
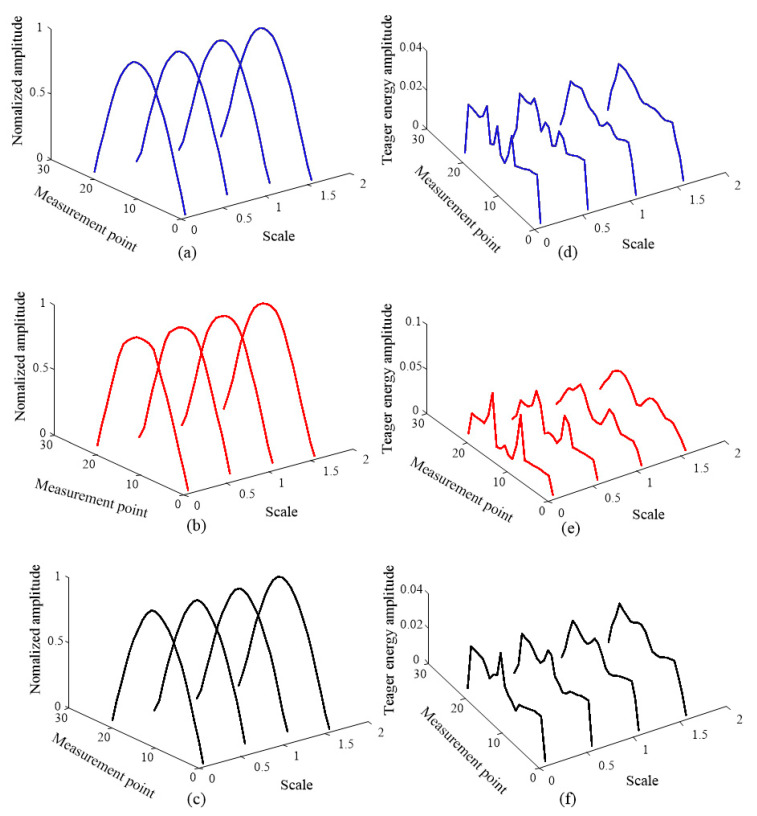
Multiscale SCDSs from simulation3: (**a**) multiscale 1× SCDSs; (**b**) multiscale 2× SCDSs; (**c**) multiscale 3× SCDSs; (**d**) multiscale 1× Teager super-harmonic characteristic deflection shapes (TSCDSs); (**e**) multiscale 2× TSCDSs; (**f**) multiscale 3× TSCDSs.

**Figure 5:**
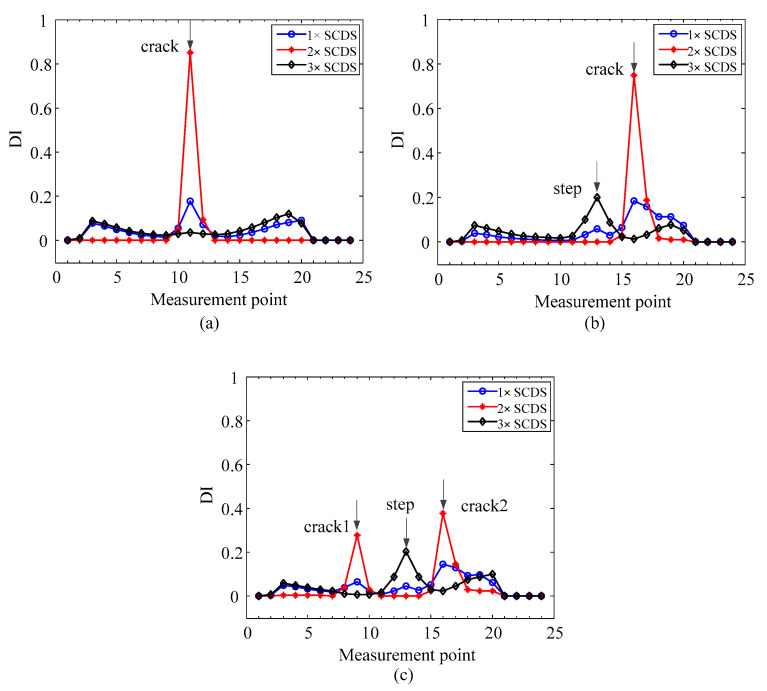
Localization results for the rotors without noise from the first three SCDSs: (**a**) simulation 1; (**b**) simulation 2; (**c**) simulation 3.

To study the performance of the crack localization method, the proposed method is applied to the simulation cases in Table 1. Firstly, the first three SCDSs without noise are extracted and shown in Figure 3. Second, the multiscale SCDSs are calculated by GMA. The scale ranges from 0 to 2 with a step of 0.1. The obtained multiscale SCDSs from simulation 3 are shown in Figure 4a–c. Then the multiscale TSCDSs are computed by applying TEO to the multiscale SCDSs. The corresponding multiscale TSCDSs from simulation 3 are shown in Figure 4d–f. Afterwards, the proper scales are adaptively selected, and the corresponding multiscale TSCDSs are fused by D-S evidence theory, and the localization results are presented in Figure 5.

From Figure 3, it can be seen that the distortions induced by cracks are more observable in the 2× SCDSs. After transforming into a multiscale space and applying the TEO, from the multiscale TSCDSs shown in Figure 4, one can see both the crack and step induced distortions are amplified for multiscale 1× TSCDSs and 3× TSCDSs, while for the multiscale 2× TSCDSs the step induced distortions are not that obvious compared to crack induced distortions. Therefore, multiscale 2× TSCDSs will perform better in crack localization under the interference of steps, which has been validated further in Figure 5 using the proposed DI with D-S evidence fusion method. In Figure 5, peaks only appear at the actual crack locations when using the multiscale 2× TSCDSs, and interference of steps has been circumvented. However, peaks are not only present at crack locations but also at step locations when using the multiscale 1× and 3× TSCDSs, which indicates that the steps interference cannot be eliminated, and that it will be misleading if no prior step information is available. Therefore, in the following investigation, only the 2× SCDSs will be considered.

To evaluate the robustness of the proposed method, rotors in simulations 1–3 under different noise levels are investigated for crack localization by using 2× SCDSs. As is known, the SCDSs extraction by FDD are able to reduce noise to some degree. Therefore, to emphasize the contribution of multiscale data fusion in improving the robustness of the proposed method, white noise is directly added to the extracted unpolluted SCDS y, and the noise-polluted SCDS $y_{N}$ is expressed as:

(26) $$y_{N}=y+\frac{L_{N}\sqrt{\sum {\left(y_{i}-\mu \right)}^{2}}}{N}r$$

where *N* is the length of the SCDS. $L_{N}$ is the noise level ranging from 0 to 1. μ is the mean value of y. r is a random number vector obeying standard normal distribution.

**Figure 6:**
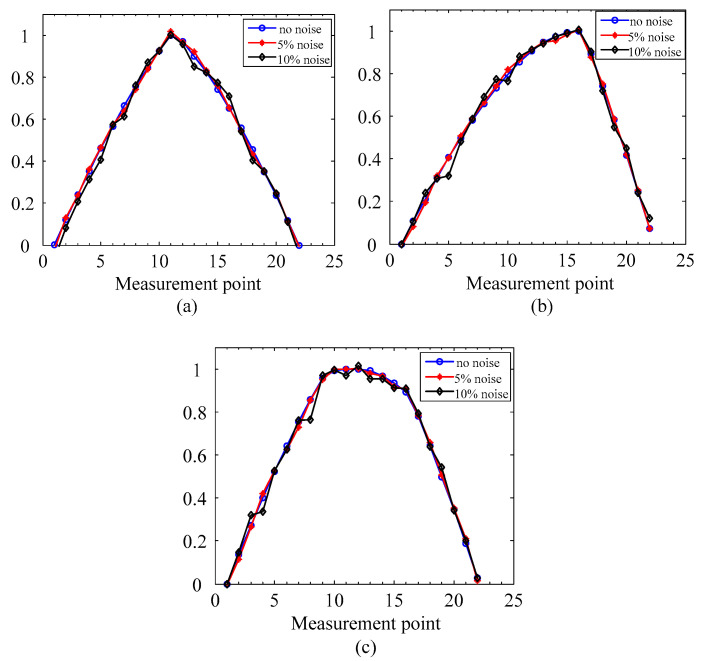
Noise-polluted 2× SCDS: (**a**) simulation 1; (**b**) simulation 2; (**c**) simulation 3.

The noise-polluted 2× SCDSs of the three simulations are shown in Figure 6. From Figure 6, when the noise level increases to 10%, the polluted SCDSs contain some obvious distortions, and it makes it difficult to localize cracks under such conditions. Therefore, we evaluate the robustness of the crack localization method with those SCDSs.

**Figure 7:**
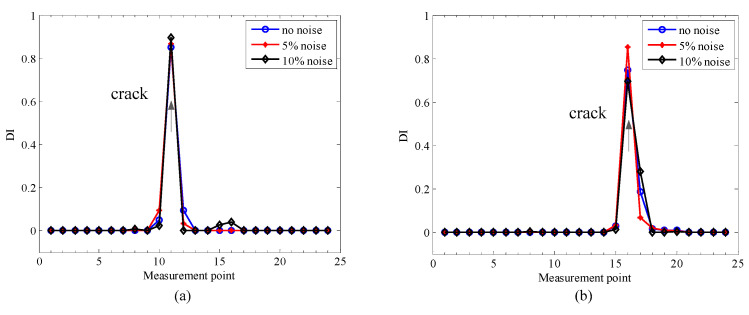

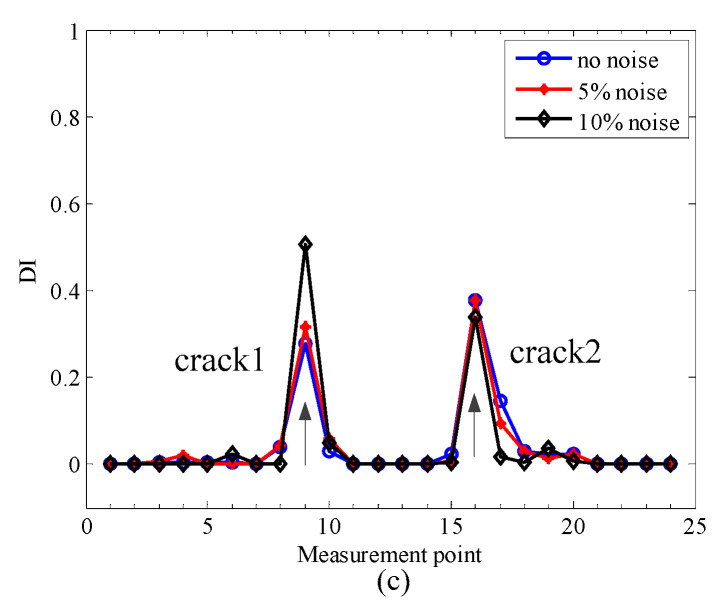
Localization results for the numerical rotors with different noise levels: (**a**) simulation 1; (**b**) simulation 2; (**c**) simulation 3.

Based on the noise-polluted 2× SCDSs, the proposed crack localization method is applied to these, and Figure 7 presents the results of crack localization under different levels of noise.

It can be seen from Figure 7 that two clear peaks are presented at real crack locations, but no interference from the steps can be found. Moreover, the crack localization accuracy is not affected by the level of noise, which indicates the good performance of the proposed method.

It should be mentioned that no intact rotor simulation is considered, and the reasons are as follows. For an intact rotor, if there are no other obvious factors generating higher order frequency components, no higher order SCDSs could be extracted. Therefore, there will be no crack and no need to perform crack localization. Moreover, if there are some nonlinear factors which will generate higher order frequency components, such as nonlinear bearings or misalignment in the coupling, the 2× or higher order SCDSs can be obtained. But the effects of these nonlinearities on the SCDSs will be global, and the continuity of the SCDSs in the range of measurement points will not be affected. Therefore, the proposed method will still be effective. To sum up, by considering the properties such as linearity of an intact rotor and its smoothness of the SCDSs, the proposed method can realize crack localization without the baseline date on pristine rotors.

## 4. Experimental Validation

**Figure 8:**
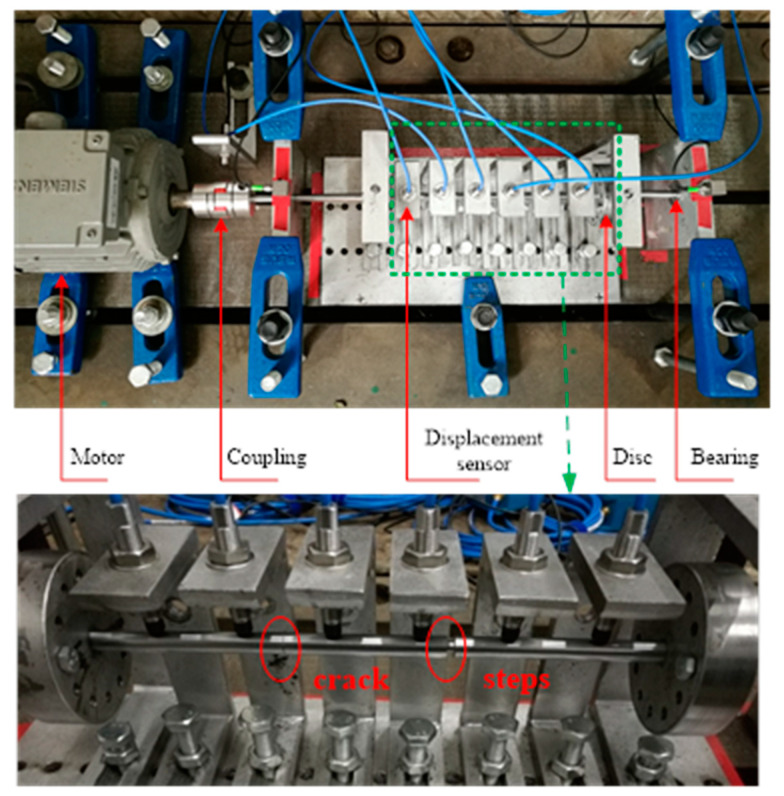
Experimental rig for crack localization.

In this section, experiments will be carried out to verify the proposed crack localization method. The test rig is shown in Figure 8, which is mainly made of the motor and its control system, coupling, under-test rotor and ball bearings. Six eddy current displacement sensors are distributed along the shaft to obtain the multiple-sensor signals in the vertical direction for real SCDS extraction. Cracks are manufactured by wire cutting. The crack width is approximately 0.2 mm. Though no breathing nonlinearity will be there in the manufactured cracks, the cracks will lead to the asymmetry of the rotor, which will induce the 2× frequency component [46], since no other obvious asymmetry factors are involved in the experimental rotor, the crack could be exclusively localized by using the 2× SCDS with the proposed method. Therefore, the crack localization method can be validated by these fabricated cracks. Three crack depths are created for the under-test rotors, which are 1.57 mm (0.157*D*), 1.54mm (0.154*D*) and 3.29 mm (0.329*D*), respectively. The step shaft is simulated by a ring slot. Detail parameters of the cracks and steps in the experimental rotors are tabulated in Table 2. The rotating speed is set at 600 r/min to ensure that the 2× frequency component is separate from the resonate frequency. The sampling frequency is chosen as 5000 Hz. To simulate the industry environment, the distribution of sensors is not strictly uniform, and no strict alignment of the rotor is performed, which more or less misaligns the under-test rotors.

**Figure 9:**
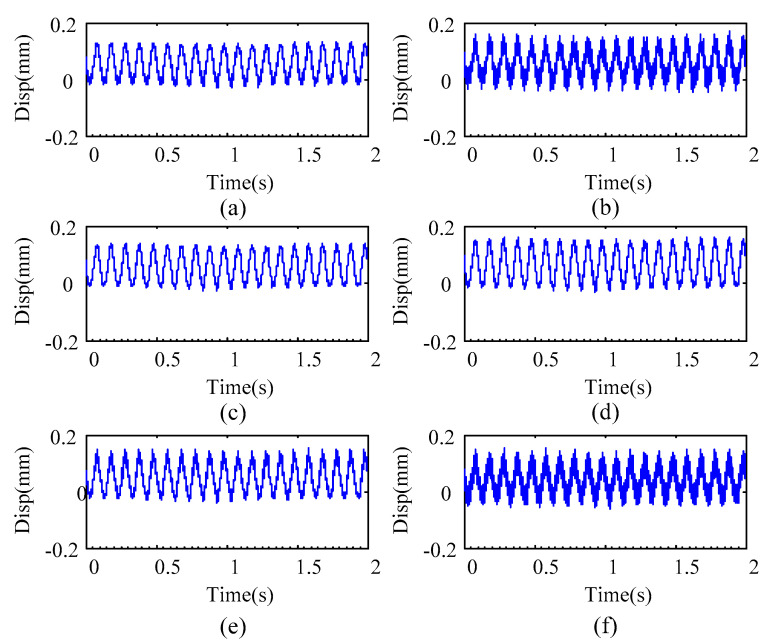
Typical measured responses from different measurement points of the rotor in case 2: (**a**) #1; (**b**) #2; (**c**) #3; (**d**) #4; (**e**) #5; (**f**) #6.

**Figure 10:**
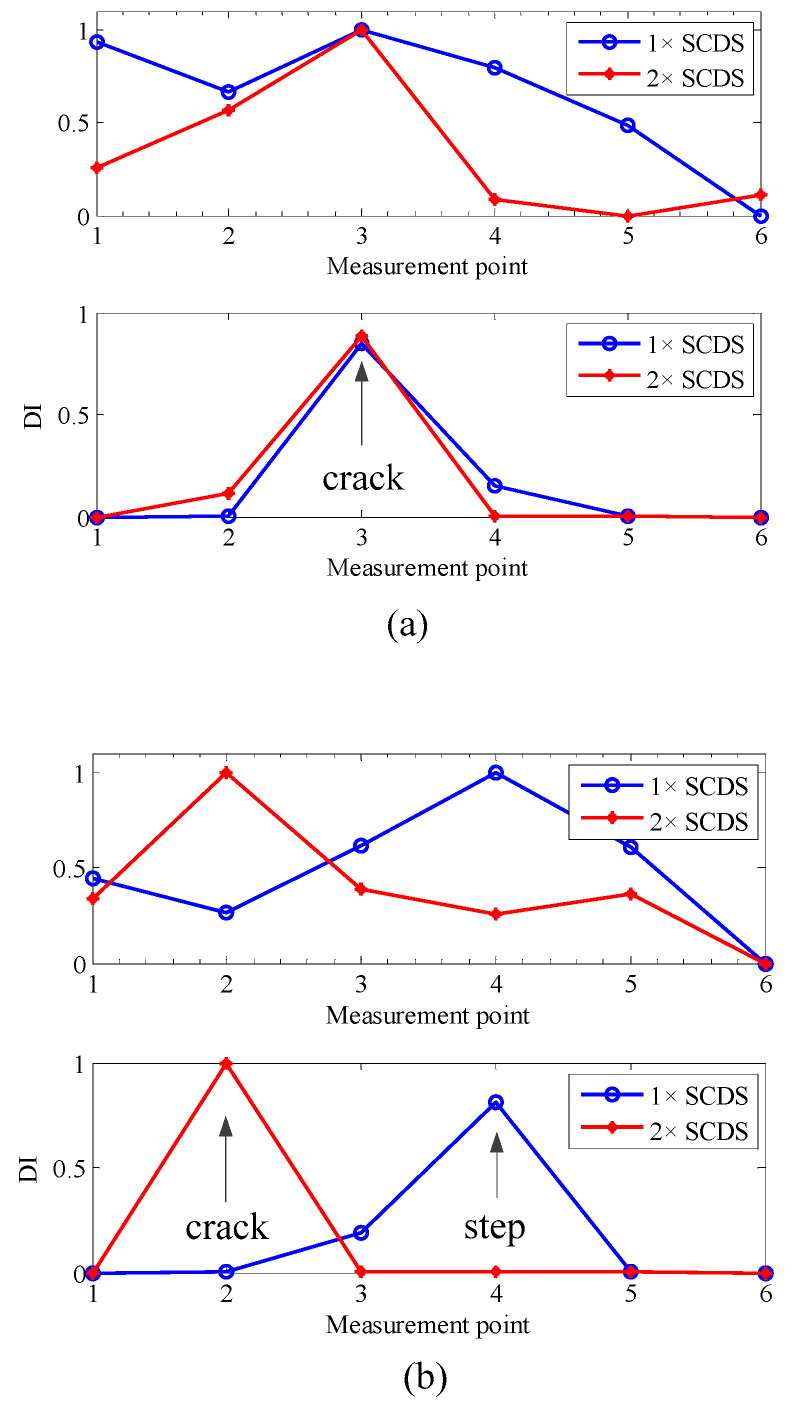

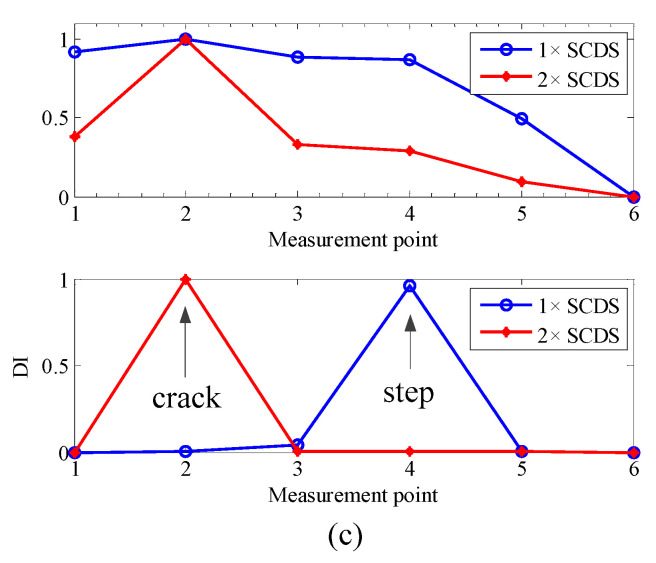
Experimental results of crack localization by the proposed method: (**a**) case 1; (**b**) case 2; (**c**) case 3.

Figure 9 presents the typical vibration responses measured from the rotor in case 2. From Figure 9, one can see that the measured responses are contaminated by noise, and a robust crack localization method is demanded in these conditions. Because the nonlinearity induced by crack asymmetry mainly appears in the 2× component, only the 1× and 2× SCDSs are considered. For applying the proposed method to those measured multiple-sensor responses for the three rotor cases, the localization results are shown in Figure 10.

For the rotor without steps in case 1, the localization results can be seen in Figure 10a. From the results, one can see that when there are no steps, the localization results based on both multiscale 1 × TSCDS and 2 × TSCDS by the proposed method are accurate. For the rotor with steps in case 2, the localization results are demonstrated in Figure 10b. It can be seen that when the proposed method is applied to 1 × SCDS, then an obvious peak is observed only at the location of the step other than the real crack location. The results indicate that the crack localization is misleading and has failed under the interference of steps. However, when utilizing 2 × SCDS for crack localization, no peak can be seen at the step location and can rather be seen at the crack location. Hence, crack localization based on 2 × SCDS by the proposed method is accurate under the interference of steps. When the crack depth increases from 1.54 mm to 3.29 mm for the rotor in case 3, similar results are obtained in Figure 10c, which manifests the crack depth robustness of the proposed method.

From the experimental results above, the conclusion can be drawn that the proposed method is effective and robust against noise for crack localization in stepped rotors based on D-S evidence fusion of multiscale 2 × TSCDSs, and shows promise in real applications.

## 5. Discussion

### 5.1. Effect of Scale Parameters

**Figure 11:**
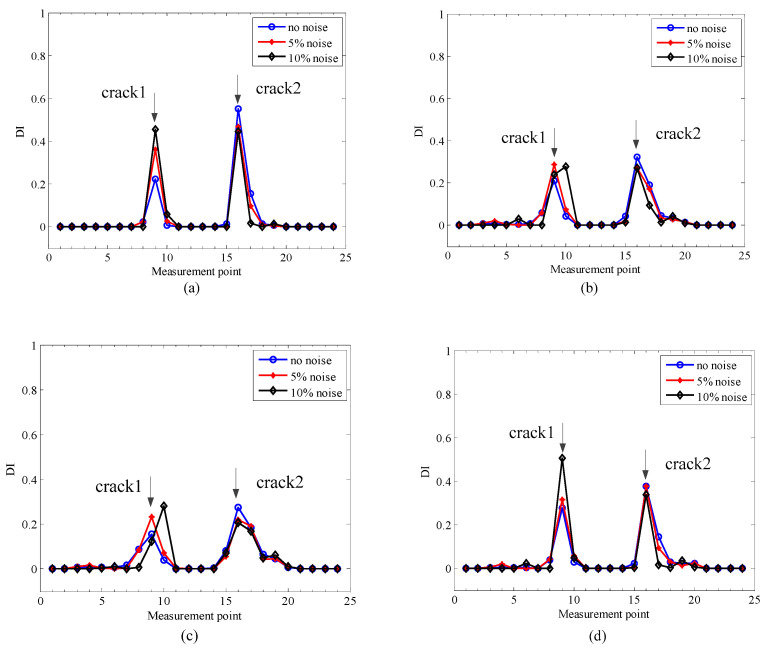
Effects of scales selected on localization results: (**a**) scale case 1; (**b**) scale case 2; (**c**) scale case 3; (**d**) adaptively selected scales.

To show the necessity and superiority of the method by adaptively selecting scales for crack localization, three different scale parameter intervals are manually selected for crack localization. The three scale intervals are [0.6, 1.5], [0.8, 1.2] and [1, 1.4] with a step size of 0.1, named scale cases 1 to 3, where scale case 1 is the whole scale interval after ignoring the boundary of the first five and last five scales. The above three scale intervals corresponding multiscale TSCDSs are fused to localize the cracks in simulation 3 under different noise levels, and their localization results are demonstrated in Figure 11a–c respectively, which are compared to the proposed method with adaptively selected scales, as shown in Figure 11d.

From Figure 11 one can see that using different scales of multiscale TSCDSs will affect the localization results especially in heavy noise condition, and the crack localization results are more accurate and robust when using the proposed adaptive scale selection method.

### 5.2. Comparison with a Single-Scale Crack Localization Method

In order to demonstrate the advantages of the proposed method, a single-scale crack localization method by gapped smoothing method (GSM) [36] with 2 × SCDS is compared, which will be called GSM method in the following. The principle of the GSM method is to detect the singularities induced by cracks in the SCDS by gapped polynomial curve fitting. The damage index in the GSM method is constructed as the squared difference between the gapped polynomial function and the value of the extracted SCDS.

**Figure 12:**
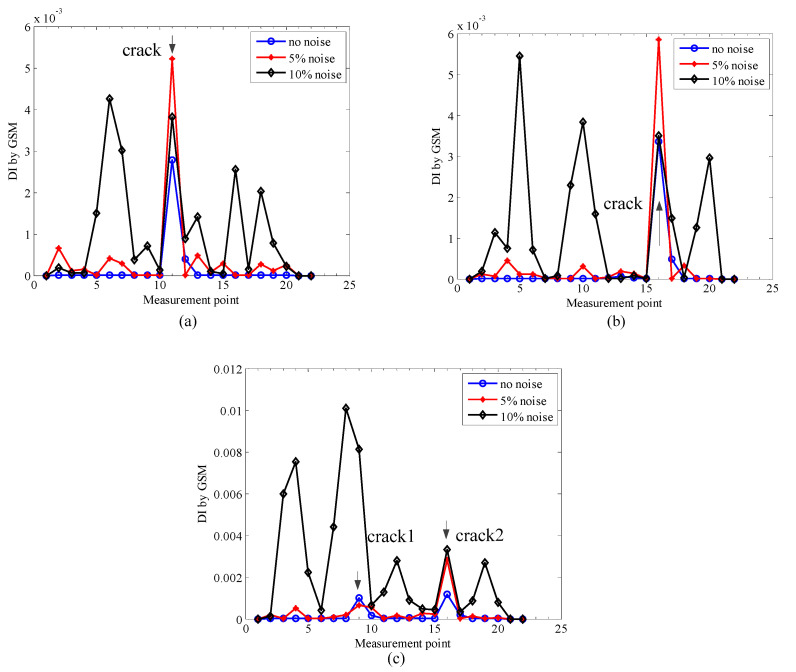
Localization results by gapped smoothing method (GSM) method under different noise levels: (**a**) simulation 1; (**b**) simulation 2; (**c**) simulation 3.

To perform the comparison, the GSM method is applied to the same rotors in the three simulations in Table 1, the localization results from the three simulations with different noise levels are shown in Figure 12.

From Figure 12, one can see that the localization results are accurate by the GSM method for the three simulation cases under no noise conditions. However, when the noise level increases to 5%, more fluctuations appear, and furthermore, when the noise level increases to 10%, the crack locations can hardly be identified. Compared with the results of the proposed localization method, which can be seen in Figure 7, although the GSM method also performs well under no noise or light noise conditions, the proposed method performs much better in heavy noise conditions, which means the proposed method is more robust in terms of noise and more suitable for real applications.

## 6. Conclusions

A novel baseline-free adaptive crack localization method for operating stepped rotors based on D-S evidence fusion of multiscale super-harmonic characteristic deflection shapes (SCDSs) was proposed. In the proposed method, the cracks were localized exclusively under the commonly existing interference of steps in the shaft, by extracting the singularities on the SCDSs induced by cracks only. The robustness of the method was improved by transforming the SCDSs into multiscale TSCDSs with Gaussian multiscale analysis and a Teager energy operator. Then, an adaptive scale selection method with fractal dimension was developed to select the proper multiscale TSCDSs. After that, the selected proper multiscale TSCDSs were used to construct a new damage index by D-S evidence fusion theory for crack localization. Both numerical and experimental investigations validated the effectiveness, accuracy and robustness of the proposed method. The comparison with a single-scale-based gapped smoothing method indicated the superiority of the proposed method in heavy noise condition.

The proposed method is baseline-free, output-only and scale selection adaptive, and the interference from the commonly existing steps can be eliminated, which would be helpful for real applications. The resolution of localization results depends on measurement points, and so the higher the resolution demanded, the more measurement points are required, which should be balanced when the proposed method is applied in practice.

## Figures and Tables

**Table 1:** Locations of cracks and steps considered in numerical simulations.

Simulations	Crack 1 (Measurement Point)	Crack 2 (Measurement Point)	Stepped Shaft (Measurement Point)
1	10–11	--	--
2	15–16	--	12–13
3	8–9	15–16	12–13

**Table 2:** Crack and step parameters of experimental rotor cases.

Case	Crack Location (Measurement Point)	Crack Depth (mm)	Step Location (Measurement Point)
1	3–4	1.57	--
2	2–3	1.54	4–5
3	2–3	3.29	4–5
